# A variant form of acute reversible cardiomyopathy: a case report

**DOI:** 10.1186/1752-1947-2-74

**Published:** 2008-03-07

**Authors:** Apostolos Karavidas, Sofia Arapi, John Fotiadis, Achilleas Zacharoulis, Evagellos Matsakas

**Affiliations:** 1 Cardiology Department, 'G. Gennimatas' Hospital, Athens, Greece

## Abstract

**Introduction:**

Stress cardiomyopathy, also known as Takotsubo cardiomyopathy or left ventricular apical ballooning, has been linked to emotional or physical stress resulting in transient left ventricular dysfunction. It typically affects the mid and apical left ventricular segments. At onset, it resembles acute myocardial infarction, due to the acute onset of chest pain and ST-T segment elevation. However, there is minimal biomarker elevation and a normal coronary artery angiogram.

**Case presentation:**

We report a case of a woman with transient myocardial injury after a stressful event, presenting with a variation of the affected segments. In this case, only the basal and mid portions of the left ventricle were affected, while the apex was completely spared. Coronary angiography revealed no significant occlusion and left ventricular function had recovered completely by the third day of hospitalization.

**Conclusion:**

We present a variant form of stress cardiomyopathy, affecting the basal and mid segments of the left ventricle.

## Introduction

Takotsubo cardiomyopathy or left ventricular (LV) apical ballooning consists of acute onset of transient akinesia, affecting the apical and mid portions of the left ventricle, accompanied by reversible, dynamic ST-T segment abnormalities, chest pain and slightly increased cardiac enzymes, without significant coronary artery stenosis. This kind of acute reversible heart injury syndrome has been named after the elective LV apical dysfunction.

We report a case comprising the characteristics of stress cardiomyopathy with a variation of the affected LV segments, sparing completely the LV apical segment.

## Case Presentation

A 64-year-old woman with a history of hypertension and hypercholesterolemia under treatment, presented to our emergency department with acute onset of substernal chest pain radiating to the neck and jaw. The pain had emerged 2 hours earlier when she had experienced near-drowning and fear of imminent death.

Physical examination on admission revealed a heart rate of 100 bpm, her blood pressure was 150-90 mmHg and her oxygen saturation was 97%. A grade 1–2/6 systolic murmur and a fourth heart sound were heard.

**Figure 1:**
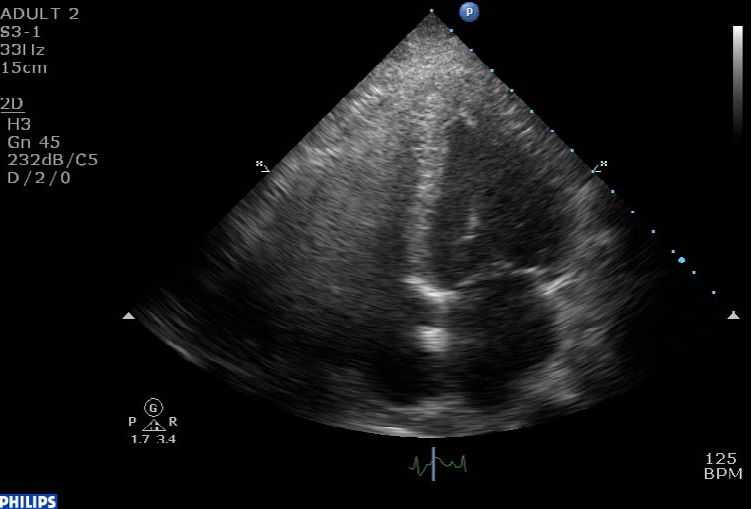
akinesis of the basal and mid segments of the ventricular septum and the lateral wall in an end-systolic 4 chamber view.

**Figure 2:**
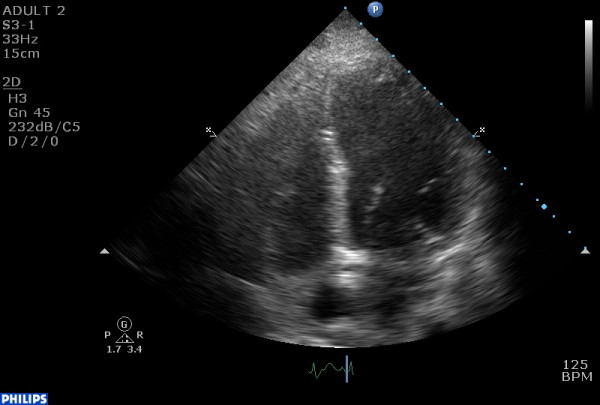
end-diastolic 4 chamber view of the left ventricle.

**Figure 3:**
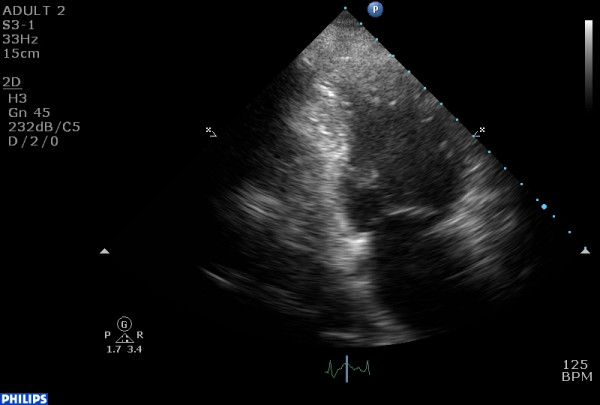
akinesis of the basal and mid segments of the anterior and posterior wall of the left ventricle in a 2 chamber end-systolic view.

ECG demonstrated ST-segment elevation in leads V2–V6. Echocardiographic evaluation depicted decreased LV ejection fraction (40%), with new regional wall motion abnormalities, i.e. hypokinesis of the basal and mid segments of the LV (fig. 1, 2, 3). It was noted that these changes had not been noted on a previous routine echocardiogram. However, subsequent coronary angiography (CAA) revealed neither major atherosclerotic lesions nor coronary spasm, while throughout her hospitalization there was only a slight increase of troponin I (cTnI) levels. Magnetic resonance imaging (MRI) was performed and showed no evidence of myocarditis. The patient had no increased inflammatory markers. No endocrine diseases or other serious concomitant disorders were present.

During her hospitalization the patient remained asymptomatic. Ventricular systolic function recovered completely and the wall motion abnormalities resolved by the 3^rd ^day following admission. Her ECG evolution showed T-wave inversion. The patient was discharged after six days, under medical treatment with a b-blocker and an angiotensin converting enzyme inhibitor. The prior occurrence of a stressful event, the normal CAA findings accompanied by cTnI levels that were disproportionate to the extent of hypokinesia and, finally, the fast LV recovery, lead us to believe that our patient had experienced a variant form of acute reversible stress cardiomyopathy.

During the next two months the patient experienced two more episodes of prolonged chest pain, both after emotional stress, bearing the same characteristics as before. However, the echocardiogram on both these occasions depicted no akinetic segments. There were neither abnormal ECG findings nor cTnI elevation.

To-date, she remains asymptomatic, with normal LV systolic function and VO2max.

## Discussion

Stress cardiomyopathy, or transient LV apical ballooning, has been described in the literature as a cardiac syndrome comprising transient LV dysfunction with chest symptoms and ECG changes mimicking those of an acute myocardial infarction. Transient LV apical ballooning on echocardiogram or ventriculography is accompanied by minimal biomarker elevations and absence of acute occlusive coronary artery disease [1-3].

In contrast to most cases reported in the literature, we report a case of stress-induced cardiomyopathy with focal wall motion abnormalities affecting only the basal and mid LV segments, sparing completely the apex, but comprising all the remaining characteristics of stress cardiomyopathy.

Various psychological and physical conditions have been reported as triggering factors of stress cardiomyopathy, including sudden accidents, stress or enhanced sympathetic activity, surgery, opioid agonist withdrawal [4] and critical illness in Intensive Care Unit patients [5].

The suggested etiology includes myocardial toxicity from catecholamines or neurogenic stunning of the myocardium after emotional or physical stress, microvascular dysfunction and multiple coronary spasm [6]. However, the results of catecholamine assays in patients with Takotsubo syndrome have varied widely and various facts call the theory of catecholamine excess in question [7].

The syndrome seems to have a predilection for women. There is a significant hemodynamic compromise in ≥ 35% of patients. The overall prognosis, however, seems to be favourable [1-3].

Our patient experienced an extremely rapid recovery of left ventricular systolic dysfunction. However, she had two recurrences of chest pain, without the full-blown clinical presentation of stress cardiomyopathy, both after experiencing severe psychological stress and while under treatment with antiadrenergic and neurohormonal antagonists. Whether or not these episodes were indicative of possible subclinical recurrences of stress cardiomyopathy, possibly averted by the received treatment, is a matter of interest. Despite the fact that the short-term prognosis of the syndrome seems to be favourable, its long-term prognosis, icluding the significance of possible recurrences, remains to be seen.

## Conclusion

Stress cardiomyopathy consists of transient LV myocardial injury, following either physical or emotional stress, mimicking acute myocardial infarction at onset, but with no significant cardiac biomarker elevation, and in the absence of acute coronary artery occlusion.

Although the LV segments usually affected are the mid and apical ones, that is not always the case and one should bear this in mind when faced with a patient with acute onset of chest pain, accompanied by transient LV dysfunction, ST-T segment elevation and a normal angiogram.

## Competing interests

The author(s) declare that they have no competing interests.

## Authors' contributions

All authors contributed substantially to the manuscript. AK and SA were involved in the care of the patient, in the acquisition and interpretation of the data and drafted the manuscript. AZ contributed to the acquisition and interpretation of the data. JF contributed to the acquisition of the data and revised the manuscript for important intellectual content. EM revised the manuscript for important intellectual content. All authors approved the final version submitted for publication.

## Consent

Written informed consent was obtained from the patient for publication of this case report and any accompanying images. A copy of the written consent is available for review by the Editor-in-Chief of this journal.
